# Endoscopic resection for non-ampullary duodenal subepithelial lesions: a retrospective cohort study

**DOI:** 10.1007/s00384-024-04698-5

**Published:** 2024-07-31

**Authors:** Yuting Jiang, Zeliang Yang, Caihao Lin, Jie Yang, Xiaoling Zheng

**Affiliations:** 1 Shengli Clinical Medical College of Fujian Medical University, Fuzhou, China; 2 Digestive Endoscopy Centre, Fujian Provincial Hospital, Fuzhou, China; 3 Fuzhou University Affiliated Provincial Hospital, Fuzhou, China

**Keywords:** Duodenum, Subepithelial lesions, Endoscopic submucosal dissection, Pre-cutting endoscopic mucosal resection, Endoscopic ultrasound

## Abstract

**Purpose:**

This study aimed to assess the safety and efficacy of endoscopic submucosal dissection (ESD) and pre-cutting endoscopic mucosal resection (pEMR) in treating non-ampullary duodenal subepithelial lesions (NADSELs) and to evaluate the clinical utility of endoscopic ultrasound (EUS) before endoscopic resection (ER).

**Methods:**

In this retrospective single-centre cohort study, we compared the clinical outcomes of patients with NADSELs who underwent ESD or pEMR between January 2014 and June 2023. The accuracies of EUS in determining the pathological type and origin of the lesions were evaluated using postoperative histopathology as the gold standard.

**Results:**

Overall, 56 patients with NADSELs underwent ER in this study, including 16 and 40 treated with pEMR and ESD, respectively. There were no significant differences between the two groups in terms of en bloc resection rate, complete (R0) resection rate, perioperative complication rate, and postoperative hospital length of stay (*P* > 0.05). However, the pEMR group had significantly shorter median operational (13.0 min vs. 30.5 min,* P* < 0.001) and mean fasting (1.9 days vs. 2.8 days, *P* = 0.006) time and lower median hospital costs (¥12,388 vs. ¥19,579, *P* = 0.002). The accuracies of EUS in determining the pathological type and origin of the lesions were 76.8% and 94.6%, respectively, compared with histopathological evaluation.

**Conclusions:**

EUS can accurately predict the origin of NADSELs. Suitable lesions determined to originate from the submucosa or more superficial layers using EUS can be treated using pEMR as it shortens the operational and recovery time, reduces hospitalisation costs, and achieves an R0 resection rate similar to ESD.

**Supplementary Information:**

The online version contains supplementary material available at 10.1007/s00384-024-04698-5.

## Introduction

Gastrointestinal subepithelial lesions (SELs) are tumours originating from the muscularis mucosa, submucosa, or muscularis propria of the gastrointestinal tract [1]. With the widespread use of endoscopic screening, the detection rate of duodenal SELs has increased. The European Society of Gastrointestinal Endoscopy [1] recommends endoscopic ultrasound (EUS) as the best tool for determining the characteristics of SELs, including size, location, layer of origin, echogenicity, and shape. A study [2] demonstrated that 19% of duodenal SELs exhibited potential malignancy, while an additional 5% were confirmed to be malignant. Considering the risk of progression, monitoring costs, and patient compliance factors, several guidelines [3–5] advocate duodenal SEL resection to obtain a histological diagnosis, irrespective of tumour size. However, conventional surgical methods are not only associated with a large wound surface, long operation and hospitalisation time, high rate of complications, and slow recovery rates, but also affect patients’ quality of life.

Owing to recent advancements in minimally invasive endoscopic treatment technologies and endoscopic suture instruments, the potential application of minimally invasive endoscopic treatment for duodenal SELs has garnered attention. Common endoscopic treatment options include endoscopic submucosal dissection (ESD), endoscopic full-thickness resection (EFR), and endoscopic mucosal resection (EMR). ESD and EFR are intricate and high-risk procedures, whereas EMR is simple and easy to perform; however, it has a limited depth of resection. Pre-cutting EMR (pEMR) is a circumferential pre-incision technique that combines the characteristics of ESD and EMR, thereby offering unique advantages. To date, few controlled studies have compared the different endoscopic resection (ER) methods for non-ampullary duodenal subepithelial lesions (NADSELs). Therefore, we aimed to assess the safety and efficacy of ESD and pEMR in the treatment of NADSELs and to evaluate the clinical utility of EUS before ER.

## Material and methods

### Patients

**Fig. 1:**
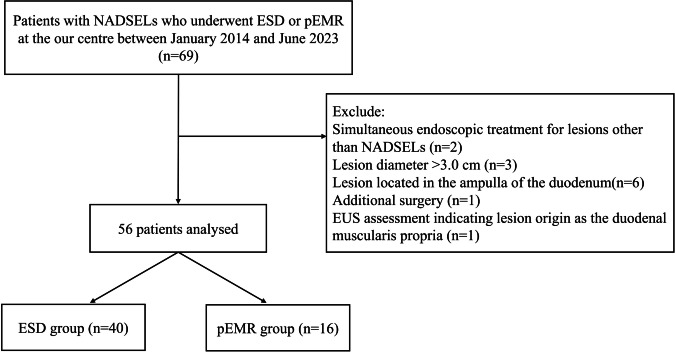
Flow chart of patients in this study. NADSELs, non-ampullary duodenal subepithelial lesions; ESD, endoscopic submucosal dissection; pEMR, pre-cutting endoscopic mucosal resection

This retrospective cohort study enrolled 56 patients with NADSELs who underwent ESD or pEMR at the digestive endoscopy centre of Fujian Provincial Hospital between January 2014 and June 2023. The inclusion criteria were as follows: (1) EUS assessment indicating lesion origin as the duodenal muscularis mucosa or submucosa; (2) EUS assessment showing that the lesions were mainly intraluminal; and (3) availability of complete hospitalisation data, including follow-up data. Exclusion criteria included any of the following: (1) simultaneous endoscopic treatment for lesions other than NADSELs; (2) recent use of antithrombotic drugs or severe coagulopathy; (3) poor general condition or severe heart and lung disease, rendering the patient unable to tolerate surgery; (4) lesion located in the ampulla of the duodenum; (5) lesion diameter > 3.0 cm; and (6) additional surgery performed because of a failed ER during the same hospitalisation period. A flowchart of the study is shown in Fig. 1. This study was approved by the Ethics Committee of the Fujian Provincial Hospital (K2023-12–009). This study adhered to the STROBE guidelines (supplementary material).

### Endoscopic therapy

Routine preoperative examinations were performed to exclude patients with contraindications. We performed EUS and upper abdominal computed tomography (CT) scan to determine lesion size, growth pattern, origin, and metastasis. The patients and their family members were fully informed, and their consent was obtained.

All patients underwent ER under general anaesthesia with continuous monitoring of vital signs, including heart rhythm, blood pressure, and blood oxygen levels during the operation. All operations were performed by experienced chief surgeons (with > 20 years of endoscopy experience and > 200 ESDs performed per year). ER was performed as follows:

ESD: (1) A dual knife (KD–650L, Olympus, Tokyo, Japan) was used for circumferential electrocoagulation labelling of the lesion periphery; (2) thereafter, multipoint submucosal injections were administered at the marked point; and (3) a circumferential incision was made, and submucosal dissection was performed using various knives (KD–650L and KD–611L, Olympus, Tokyo, Japan) until complete lesion removal was achieved.

pEMR: The labelling and submucosal injection processes were the same as those performed during ESD. After administering the submucosal injection, a circumferential submucosal incision was made to achieve sufficient resection depth, followed by an immediate submucosal injection for full lesion elevation. Thereafter, lesion resection was performed using a snare (AG–5071–241523, AGS MedTech, Hangzhou, China) by applying a high-frequency current.

After lesion resection, an electric coagulation forceps was used to achieve haemostasis at both the wound surface and its edges. If necessary, haemostatic clips or purse-string suturing with metal clips combined with a nylon rope were used.

### Postoperative follow-up

All patients underwent postoperative endoscopic examinations after 6 and 12 months and thereafter annually. For patients with incomplete resection, an abdominal CT scan was performed to evaluate recurrence or metastasis. Patients underwent regular follow-ups every 3–6 months through telephone calls, outpatient visits, and online platforms until 31 December 2023.

### Observation indicators and definitions

Adverse events included the following: (1) postoperative infection, defined as a postoperative body temperature exceeding 37.5 °C and/or increased levels of inflammatory indicators, such as blood levels of C-reactive protein or calcitonin; and (2) intraoperative perforation, defined as a defect in the serosa of the duodenum during the operation.

ER efficacy evaluation indicators were as follows: (1) en bloc resection in which the lesion was resected en bloc and a single specimen was obtained after the operation; (2) complete resection (R0 resection) defined as the absence of residual tumour at the resection margin; (3) incomplete resection (R1 resection) defined as the absence of macroscopic tumour residue at the resection margin but visible under the microscope.

### Statistical analysis

Statistical software (SPSS 25.0) was used to analyse the data. Qualitative data were expressed as frequency (percentage), quantitative data were expressed as mean (‾x ± s), and data that did not follow a normal distribution were expressed using median (M) and inter-quartile range (Q1, Q3). A two-sample *t*-test or Mann–Whitney U test was used for comparing quantitative data between the two groups, and the chi-square test or Fisher’s exact test was used for comparing qualitative data, with a two-sided *P* < 0.05 considered statistically significant.

## Results

### Baseline clinicopathological characteristics

A total of 56 patients with NADSELs (33 males and 23 females) with an average age of 56 years (56.5 ± 10.9) were enrolled. The lesions were located in the duodenal bulb (*n* = 42), duodenal bulbar descending junction (*n* = 1), or descending duodenum (*n* = 13). The median lesion diameter was 1.0 cm (0.3–3.0 cm). The pathology of the resected cases varied, with final diagnoses of Brunner’s adenoma (*n* = 24), neuroendocrine tumour (*n* = 22), ectopic pancreas (*n* = 3), lipoma (*n* = 2), gastrointestinal stromal tumour (*n* = 2), leiomyoma (*n* = 1), inflammatory fibroid polyp (*n* = 1), and gangliocytic paraganglioma (*n* = 1).

Of the 56 patients, 40 underwent ESD and 16 underwent pEMR. There were no significant differences in age, sex, location, layer of origin assessed using EUS, or tumour size between the ESD and pEMR groups (*P* > 0.05) (Table 1). Additionally, 54 lesions were determined using EUS to originate from the submucosa. Among these, 71.8% (28/39) and 80.0% (12/15) of the lesions in the ESD and pEMR groups had clear boundaries between the submucosa and muscularis propria, and there was no significant difference between the two groups (*χ*^2^ = 0.073, *P* = 0.787).

**Table 1:** Comparison of baseline characteristics of NADSELs between ESD and pEMR groups

Item	ESD group (*n* = 40)	pEMR group (*n* = 16)	*P* value
Age (years)	56.7 ± 11.0	55.9 ± 11.2	0.810
Sex			0.797
Male	24 (60.0)	9 (56.3)	
Female	16 (40.0)	7 (43.7)	
Location			0.146
Bulb	32 (80.0)	10 (62.5)	
Bulbar descending junction	0 (0.0)	1 (6.2)	
Descending duodenum	8 (20.0)	5 (31.3)	
Origination (preoperative EUS)			0.494
Mucosal muscle	1 (2.5)	1 (6.2)	
Submucosa	39 (97.5)	15 (93.8)	
Size (cm)	1.0 (0.7, 1.2)	0.9 (0.6, 1.2)	0.571

*ESD* endoscopic submucosal dissection, *pEMR* pre-cutting endoscopic mucosal resection, *EUS* endoscopic ultrasound

### Comparison of outcomes and adverse events

**Fig. 2:**
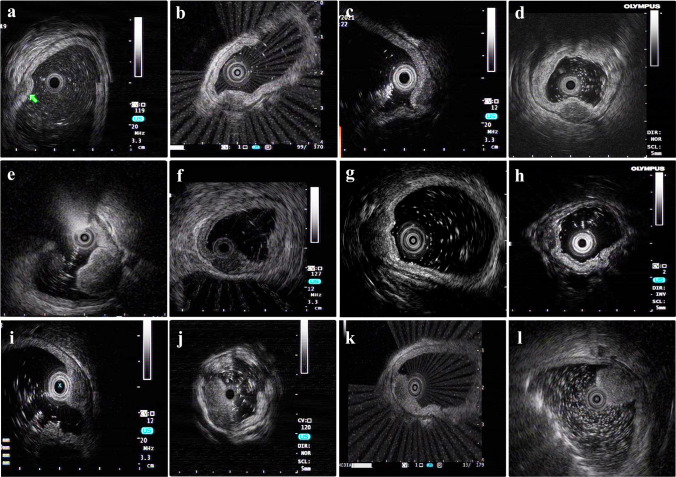
Endoscopic ultrasound images of patients who underwent R1 resection. Note: **a**–**j** Boundary between the submucosa and muscularis propria at the lesion is not clear; **k**–**l** boundary between the submucosa and muscularis propria is clear; R1 resection, incomplete resection

No adverse events were observed in the pEMR group, whereas in the ESD group, one patient developed a postoperative infection, two developed intraoperative perforation, and another two developed both intraoperative perforation and postoperative infection. However, all five patients had good prognoses after undergoing conservative treatment, such as endoscopic suturing and anti-infective therapy. There were no significant differences between pEMR and ESD groups in terms of en bloc resection rate (93.8% vs. 92.5%, *P* = 1.000), R0 resection rate (81.2% vs. 77.5%, *P* = 1.000), adverse events rate (0.0% vs. 12.5%, *P* = 0.335), and postoperative length of hospital stay [(5.9 ± 4.9) days vs. (6.1 ± 3.2) days, *P* = 0.822]. However, the pEMR group had a significantly shorter operational and postoperative fasting time than the ESD group (median operational time: 13.0 min vs. 30.5 min, *P* < 0.001; mean postoperative fasting time: 1.9 days vs. 2.8 days, *P* = 0.006). The median hospitalisation expenses were significantly lower in the pEMR group than that in the ESD group (¥12,388 vs. ¥19,579, *P* = 0.002) (Table 2). Twelve patients underwent R1 resection, and all opted for follow-up instead of additional endoscopic or surgical resection after comprehensive risk notification. The specific clinical and pathological characteristics and prognosis of these patients are detailed in Table 3 and Fig. 2. All patients, including those who underwent R1 resection, were monitored for a median period of 31.6 months, with a range extending from 6.1 to 105.7 months. Throughout this interval, no local recurrence or distant metastasis was observed.

**Table 2:** Patients’ treatment outcomes based on treatment modalities

Item	ESD group (*n* = 40)	pEMR group (*n* = 16)	*P* value
Operational time (min)	30.5 (22.5, 49.5)	13.0 (9.0, 17.8)	** < 0.001**
Excision condition			1.000
En bloc resection	37 (92.5)	15 (93.8)	
Piecemeal resection	3 (7.5)	1 (6.2)	
Resection margin			1.000
R0 resection	31 (77.5)	13 (81.2)	
R1 resection	9 (22.5)	3 (18.8)	
Complications	5 (12.5)	0 (0.0)	0.335
Postoperative infection	3 (7.5)	0 (0.0)	0.550
Intraoperative perforation	4 (10.0)	0 (0.0)	0.460
Hospitalisation expenses (RMB)	19,579 (14,814, 23,008)	12,388 (7481, 16,923)	**0.002**
Postoperative fasting time (days)	2.8 ± 1.6	1.9 ± 0.7	**0.006**
Postoperative hospital length of stay (days)	6.1 ± 3.2	5.9 ± 4.9	0.822
Follow-up duration (months)	33.5 (17.1, 67.1)	24.4 (13.5, 75.3)	0.468
Recurrence	0 (0.0)	0 (0.0)	1.000
Metastasis	0 (0.0)	0 (0.0)	1.000
Additional surgery	0 (0.0)	0 (0.0)	1.000
Survival	40 (100.0)	16 (100.0)	1.000

*ESD* endoscopic submucosal dissection, *pEMR* pre-cutting endoscopic mucosal resection, *RMB* Renminbi (Chinese currency)

**Table 3:** Clinicopathological features and outcomes of patients who underwent R1 resection

Group	Sex	Age (years)	Location	Size (cm)	Origin (EUS)	Boundary^*^	Pathology	Recurrence or metastasis	Additional surgery	Follow-up duration (months)
pEMR	M	56	D1	0.6	Submucosa	Unclear	NET	No	No	24.0
pEMR	F	48	D2	0.6	Submucosa	Unclear	NET	No	No	15.0
pEMR	M	65	D2	1.2	Submucosa	Unclear	BA	No	No	7.7
ESD	F	67	D1	0.8	Submucosa	Unclear	NET	No	No	16.0
ESD	M	61	D1	1.2	Submucosa	Unclear	NET	No	No	82.3
ESD	F	44	D1	0.8	Submucosa	Unclear	NET	No	No	104.0
ESD	F	63	D1	1.2	Submucosa	Unclear	GIST	No	No	16.5
ESD	M	75	D1	0.5	Submucosa	Unclear	NET	No	No	24.6
ESD	M	53	D1	0.9	Submucosa	Unclear	NET	No	No	34.3
ESD	F	42	D1	1.2	Submucosa	Unclear	NET	No	No	27.0
ESD	M	71	D1	1.0	Submucosa	Clear	NET	No	No	10.0
ESD	M	63	D2	1.4	Submucosa	Clear	NET	No	No	80.6

^*^Boundary between the submucosa and muscularis propria, assessed by EUS

*ESD* endoscopic submucosal dissection, *pEMR* pre-cutting endoscopic mucosal resection, *EUS* endoscopic ultrasound, *NET* neuroendocrine tumour, *GIST* gastrointestinal stromal tumour, *BA* Brunner’s adenoma, *F* female, *M* male, *D1* bulb of the duodenum, *D2* descending part of the duodenum

### Accuracy of EUS in assessing the pathological type and origin of NADSELs

Among the 56 patients, 21 were diagnosed with Brunner’s adenomas, 28 with neuroendocrine tumours, two with gastrointestinal stromal tumours, three with lipomas, and two with ectopic pancreas using EUS. Among them, 13 patients were misdiagnosed and five cases of Brunner’s adenoma were misdiagnosed as gastrointestinal stromal tumours (one case), lipomas (one case), and neuroendocrine tumours (three cases). One case of gastrointestinal stromal tumour was misdiagnosed as a neuroendocrine tumour. One case of leiomyoma was misdiagnosed as Brunner’s adenoma, one of gangliocytic paraganglioma was misdiagnosed as a neuroendocrine tumour, and two of neuroendocrine tumours were misdiagnosed as Brunner adenoma and ectopic pancreas, while one case of inflammatory fibroid polyp was misdiagnosed as a neuroendocrine tumour and two cases of ectopic pancreas were misdiagnosed as neuroendocrine tumours. In conclusion, the accuracy of EUS in diagnosing pathological NADSELs was 76.8% (43/56).

The origin of the lesions was assessed using EUS, which showed that two cases originated from the muscularis mucosa and 54 from the submucosa. Postoperative pathological evaluation revealed that two, 53, and one lesion originated from the muscularis mucosa, submucosa, and muscularis propria, respectively. The accuracy of EUS in assessing the origin of the SELs was 94.6% (53/56) (Table 4).

**Table 4:** Comparing lesion’s origin assessment between EUS and postoperative histopathology

EUS	Postoperative histopathological			Total
	Muscularis mucosa	Submucosa	Muscularis propria	
Muscularis mucosa	1	1	0	2
Submucosa	1	52	1	54
Total	2	53	1	56

*EUS* endoscopic ultrasound

## Discussion

ER of duodenal lesions remains a challenging procedure because of the unique and intricate anatomical and physiological characteristics of the duodenum, including its thin muscular layer, rich blood supply, and limited endoscopic operating space. Consequently, most current studies have focused on the ER of lesions in the oesophagus and stomach [6, 7], with limited research, primarily case reports on the ER of duodenal SELs [8–10]. This study analysed and summarised the applications of ESD and pEMR for the treatment of NADSELs at our centre over the past decade. Considering that tumours located in the duodenal ampulla and those with large diameters are predictive of more invasive lesions [11–14], only non-ampullary SELs with a diameter of ≤ 3 cm were considered in this study. The results of this study indicate that pEMR is more cost-effective and efficient than ESD, with the added advantage of a faster postoperative recovery rate.

Ren et al. [15] retrospectively analysed 32 NADSELs treated with EFR. The R0 resection rate of EFR was 100%; however, in their study, one patient underwent laparotomy, another underwent laparoscopic exploration for delayed perforation after the operation, and another was transferred to the intensive care unit for further treatment due to decreased saturation of pulse oxygen. This result suggests that, although EFR can ensure complete lesion resection, its high technical requirements and risks are limiting factors against its widespread application. Moreover, because of the limited number of patients treated with EFR at our centre, NADSELs treated with EFR were not included in this study. Conventional EMR, while having low operational difficulty and high safety, often fails to achieve adequate submucosal resection for duodenal SELs that had infiltrated the submucosa or deeper layers, resulting in a high positivity rate for vertical margin in the resected lesion. In contrast, ESD, while capable of achieving sufficient submucosal dissection to obtain negative vertical margins, is associated with higher operational complexity and complication rates [16–19]. Previous reports on ESD for duodenal lesions have indicated perforation rates of 13–50% and delayed bleeding rates of approximately 20% [20, 21]. In contrast, pEMR, a method combining ESD and EMR, involves a circumferential incision similar to that in ESD to the depth of the submucosa around the target tumour, followed by a direct snare resection. Circumferential pre-cutting allows the snare to anchor to the target lesion, resulting in a more accurate resection at a depth closer to the muscularis propria [22]. Compared with ESD, pEMR significantly reduces procedural difficulty and avoids challenging, risky, and time-consuming submucosal dissections. Studies [23–26] have confirmed the safety and effectiveness of pEMR for the resection of colorectal lesions or non-ampullary superficial duodenal lesions, prompting our exploration of its suitability for NADSELs. In our study, 56 patients diagnosed using EUS with tumours originating from the submucosa and more superficial layers were included, with 40 and 16 patients undergoing ESD and pEMR, respectively. Similar to previous studies on colorectal lesions, no significant difference was observed in the incidence of complications and R0 resection rate between the two groups. However, operational time was significantly shorter in the pEMR group. Two additional evaluation indicators, postoperative fasting time and hospitalisation cost, were included; the mean postoperative fasting time and median hospitalisation cost in the pEMR group were lower than those in the ESD group.

Crucial measures for evaluating the efficacy of ER include en bloc and R0 resection rates. En bloc and R0 resection rates of 78.7–100% and 76.6–90%, respectively, have been reported for ER of duodenal SELs [18, 27–31]. In our study, the en bloc and R0 resection rates were consistent with those reported by previous studies. However, unlike the findings from previous studies [24, 25], the en bloc (93.8% vs. 92.5%) and R0 (81.2% vs. 77.5%) resection rates in the pEMR group were marginally higher than those in the ESD group, although these differences were not statistically significant. This result may be attributed to several factors. In previous studies of colorectal lesions, both ESD and pEMR allowed for specimen retrieval using a snare after tumour resection, whereas our study focused on duodenal lesions, where failure to remove the specimen immediately after tumour resection could result in its transportation by gastrointestinal peristaltic waves to the distal duodenum or small intestine, making specimen retrieval unfeasible. In the pEMR group, the specimen was immediately removed using a snare. Nevertheless, the specimen retrieval methods used in the ESD group, often involving aspiration at the gastroscope’s apex and subsequent extraction through the mouth or the gastroscope’s biopsy channel, could have led to specimen fragmentation, potentially reducing the en bloc and R0 resection rates. Moreover, pEMR involves a one-time complete resection of suitable lesions, including the capsule, whereas ESD dissection along the tumour may cause mechanical damage to the capsule and result in residual tumour cells. Additionally, a total of 12 patients in the two groups underwent R1 resection. Considering existing clinical practice guidelines [5, 32–34] do not routinely endorse re-resection for cases after receiving R1 resection, all patients chose to proceed with follow-up monitoring rather than an additional surgical treatment after our comprehensive risk notification. This decision was made with the understanding that close monitoring could effectively manage their condition without the immediate need for further invasive procedures. The vigilant postoperative follow-up yielded reassuring results: none of the patients, including those who underwent R1 resection, exhibited signs of tumour recurrence or metastasis. This outcome also suggests that the efficacy of pEMR for treating NADSELs was comparable to that of ESD.

SEL surfaces usually cover the normal mucosa. Conventional white-light endoscopy cannot provide comprehensive information regarding the nature and origin of such lesions. However, different types of SELs exhibit distinct ultrasonographic images. By observing the relationship between the lesion and its surrounding tissue, growth pattern, actual size, layers of origin, and blood supply, EUS can offer preliminary clinical diagnostic guidance and serve as a critical foundation for ER. Using histopathology as the gold standard, preoperative EUS achieved a 76.8% accuracy rate in diagnosing the pathological types of the lesions analysed in this study, which is similar to the findings of He et al. [35]. The origin and invasive level of SELs are pivotal considerations in selecting appropriate treatment methods because of variations in resection depth across different techniques. In our study, the accuracy of EUS in determining the origin of the lesion was 94.6%, indicating that it can provide valuable guidance for selecting an appropriate treatment strategy. Theoretically, both ESD and pEMR can achieve ideal therapeutic effects for lesions originating from the submucosal or superficial layers, as determined using EUS. In this study, 12 patients underwent R1 resection, and following retrospective analysis and summary of their preoperative EUS characteristics, all lesions were found to be located in the submucosa. The boundary between the submucosa and muscularis propria was indistinct before R1 resection in the pEMR group, and 77.8% (7/9) of the lesions in the ESD group exhibited an unclear boundary between the submucosa and muscularis propria before R1 resection. Our hypothesis suggests that this EUS feature may indicate the unsuitability of pEMR for such lesions; instead, ESD or EFR should be chosen to ensure complete resection. However, this hypothesis warrants further investigation.

This study has limitations. First, this was a retrospective study rather than a randomised trial, and a selection bias might have occurred. The indications for the different ER methods for NADSELs were not clearly defined and were generally based on the surgeon’s experience, although no differences in baseline characteristics were observed between the two groups. Second, the included lesions originated only from the submucosa and more superficial layers, limiting the generalisability of the study findings to lesions originating from the muscularis propria or deeper layers. Finally, the small sample size, particularly in the pEMR group, may have affected the reliability of the results. However, to the best of our knowledge, this study is one of the studies with the largest sample size to have compared the different ER methods for NADSELs.

## Conclusion

EUS has a diagnostic value for NADSELs and can be used as a guide for selecting appropriate ER procedures. When EUS shows that a lesion originates from the submucosa or more superficial layer and that the lesion diameter is ≤ 3.0 cm, pEMR should be considered as it is time-saving and efficient and can achieve a similar clinical treatment effect as ESD. However, the results of this study need further verification using prospective, multicentre, large-sample size studies.

## Supplementary Information

Below is the link to the electronic supplementary material.

Supplementary file1 (DOC 93 KB)

## Data Availability

Data available on request from the authors.

## References

[1] Deprez PH, Moons L, OʼToole D et al (2022) Endoscopic management of subepithelial lesions including neuroendocrine neoplasms: European Society of Gastrointestinal Endoscopy (ESGE) Guideline. Endoscopy 54(4):412–429 35180797 10.1055/a-1751-5742

[2] Polkowski M (2005) Endoscopic ultrasound and endoscopic ultrasound-guided fine-needle biopsy for the diagnosis of malignant submucosal tumors. Endoscopy 37(7):635–645. 10.1055/s-2005-861422 16010608 10.1055/s-2005-861422

[3] Nishida T, Hirota S, Yanagisawa A et al (2008) Clinical practice guidelines for gastrointestinal stromal tumor (GIST) in Japan: English version. Int J Clin Oncol 13(5):416–430 18946752 10.1007/s10147-008-0798-7

[4] Li J, Ye Y, Wang J, Zhang B, Qin S, Shi Y, He Y, Liang X, Liu X, Zhou Y, Wu X, Zhang X, Wang M, Gao Z, Lin T, Cao H, Shen L, Tumor CSOCOCECOGS (2017) Chinese consensus guidelines for diagnosis and management of gastrointestinal stromal tumor. Chin J Cancer Res 29(4):281–93 28947860 10.21147/j.issn.1000-9604.2017.04.01 PMC5592117

[5] Panzuto F, Ramage J, Pritchard DM, van Velthuysen MF, Schrader J, Begum N, Sundin A, Falconi M, O’Toole D (2023) European Neuroendocrine Tumor Society (ENETS) 2023 guidance paper for gastroduodenal neuroendocrine tumours (NETs) G1–G3. J Neuroendocrinol 35(8):e13306 37401795 10.1111/jne.13306

[6] Zhang Y, Wen J, Zhang S et al (2022) Clinical study of submucosal tunneling endoscopic resection and endoscopic submucosal dissection in the treatment of submucosal tumor originating from the muscularis propria layer of the esophagus. Medicine (Baltimore) 101(51):e32380 36595766 10.1097/MD.0000000000032380 PMC9794317

[7] Liu S, Zhou X, Yao Y, Shi K, Yu M, Ji F (2020) Resection of the gastric submucosal tumor (G-SMT) originating from the muscularis propria layer: comparison of efficacy, patients’ tolerability, and clinical outcomes between endoscopic full-thickness resection and surgical resection. Surg Endosc 34(9):4053–4064 32016516 10.1007/s00464-019-07311-x PMC7394934

[8] Uchima H, Garsot E, Colán-Hernández J et al (2022) Endoscopic full-thickness resection of a duodenal gastrointestinal stromal tumor with extraluminal component: the usefulness of traction and sutures. Endoscopy 54(12):E730–E731 35272380 10.1055/a-1773-0260

[9] Kim M, Bareket R, Kahaleh M (2022) Endoscopic submucosal dissection of a duodenal GI stromal tumor assisted by endoloops. Endoscopy 54(6):E316–E317 34243199 10.1055/a-1526-0199

[10] Baldaque-Silva F, Wang N, Rouvelas I, Omae M (2022) Traction-assisted endoscopic submucosal dissection of a duodenal gastrointestinal stromal tumor. Endoscopy 54(6):E318–E319 34243195 10.1055/a-1527-7600

[11] Liu M, Song C, Zhang P et al (2020) A nomogram for predicting cancer-specific survival of patients with gastrointestinal stromal tumors. Med Sci Monit 26:e922378 32449506 10.12659/MSM.922378 PMC7268888

[12] Lee SW, Sung JK, Cho YS et al (2019) Comparisons of therapeutic outcomes in patients with nonampullary duodenal neuroendocrine tumors (NADNETs): a multicenter retrospective study. Medicine (Baltimore) 98(26):e16154 31261543 10.1097/MD.0000000000016154 PMC6617016

[13] Vanoli A, La Rosa S, Klersy C et al (2017) Four neuroendocrine tumor types and neuroendocrine carcinoma of the duodenum: analysis of 203 cases. Neuroendocrinology 104(2):112–125 26910321 10.1159/000444803

[14] Gincul R, Ponchon T, Napoleon B et al (2016) Endoscopic treatment of sporadic small duodenal and ampullary neuroendocrine tumors. Endoscopy 48(11):979–986 27494453 10.1055/s-0042-112570

[15] Ren Z, Lin SL, Zhou PH et al (2019) Endoscopic full-thickness resection (EFTR) without laparoscopic assistance for nonampullary duodenal subepithelial lesions: our clinical experience of 32 cases. Surg Endosc 33(11):3605–3611 31240477 10.1007/s00464-018-06644-3

[16] Kim GH, Kim JI, Jeon SW et al (2014) Endoscopic resection for duodenal carcinoid tumors: a multicenter, retrospective study. J Gastroenterol Hepatol 29(2):318–324. 10.1111/jgh.12390 24117946 10.1111/jgh.12390

[17] Dasari B, Al-Shakhshir S, Pawlik TM et al (2018) Outcomes of surgical and endoscopic resection of duodenal neuroendocrine tumours (NETs): a systematic review of the literature. J Gastrointest Surg 22(9):1652–1658. 10.1007/s11605-018-3825-7 29869091 10.1007/s11605-018-3825-7

[18] Nishio M, Hirasawa K, Ozeki Y et al (2020) Short- and long-term outcomes of endoscopic submucosal dissection for non-ampullary duodenal neuroendocrine tumors. Ann Gastroenterol 33(3):265–271. 10.20524/aog.2020.0477 32382229 10.20524/aog.2020.0477 PMC7196614

[19] Brito HP, Torres IT, Turke KC, Parada AA, Waisberg J, Botelho RV (2021) Comparison of endoscopic resection techniques for duodenal neuroendocrine tumors: systematic review. Endosc Int Open. 9(8):E1214–E1221 34447867 10.1055/a-1487-5594 PMC8383086

[20] Kato M, Kanai T, Yahagi N (2022) Endoscopic resection of superficial non-ampullary duodenal epithelial tumor. DEN Open 2(1):e54 35310765 10.1002/deo2.54 PMC8828234

[21] Nonaka S, Oda I, Tada K et al (2015) Clinical outcome of endoscopic resection for nonampullary duodenal tumors. Endoscopy 47(2):129–135 25314330 10.1055/s-0034-1390774

[22] Moss A, Bourke MJ, Tran K et al (2010) Lesion isolation by circumferential submucosal incision prior to endoscopic mucosal resection (CSI-EMR) substantially improves en bloc resection rates for 40-mm colonic lesions. Endoscopy 42(5):400–404 20213591 10.1055/s-0029-1243990

[23] Oh CK, Lee BI, Lee SH et al (2022) Circumferential submucosal incision prior to endoscopic mucosal resection versus conventional endoscopic mucosal resection for colorectal lesions with endoscopic features of sessile serrated lesions. Surg Endosc 36(3):2087–2095. 10.1007/s00464-021-08495-x 33913030 10.1007/s00464-021-08495-x

[24] Cheung DY, Choi SK, Kim HK et al (2015) Circumferential submucosal incision prior to endoscopic mucosal resection provides comparable clinical outcomes to submucosal dissection for well-differentiated neuroendocrine tumors of the rectum. Surg Endosc 29(6):1500–1505 25277474 10.1007/s00464-014-3831-0

[25] Zhang YX, Liu X, Gu F, Ding SG (2024) Planned hybrid endoscopic submucosal dissection as alternative for colorectal neoplasms: a propensity score-matched study. Dig Dis Sci 69:949–960 38218733 10.1007/s10620-023-08195-7

[26] Chen D, Fu S, Shen J (2024) Efficacy and safety of precutting endoscopic mucosal resection versus endoscopic submucosal dissection for non-ampullary superficial duodenal lesions. Clin Res Hepatol Gastroenterol 48:102304 38367801 10.1016/j.clinre.2024.102304

[27] Kim TW, Kim GH, Park DY et al (2017) Endoscopic resection for duodenal subepithelial tumors: a single-center experience. Surg Endosc 31(4):1936–1946 27553800 10.1007/s00464-016-5200-7

[28] Geng ZH, Zhu Y, Qu YF et al (2023) Risk factors for complications and incomplete resection after endoscopic resection for duodenal submucosal tumors. Surg Endosc 37:9183–9189 37845536 10.1007/s00464-023-10455-6

[29] Manta R, Zito FP, Pugliese F, Caruso A, Mangiafico S, D’Alessandro A, Castellani D, Germani U, Mutignani M, Conigliaro RL, Bonetti LR, Matsuda T, De Francesco V, Zullo A, Galloro G (2023) Endoscopic submucosal dissection for subepithelial tumor treatment in the upper digestive tract: a western, multicenter study. GE Port. J. Gastroenterol 30(2):115–20 37008525 10.1159/000525993 PMC10050838

[30] Nabi Z, Ramchandani M, Asif S, Basha J, Chavan R, Darisetty S, Reddy N (2022) Outcomes of endoscopic submucosal dissection in duodenal neuroendocrine tumors. J Gastrointest Surg 26(1):275–277 34508292 10.1007/s11605-021-05133-8

[31] Gupta S, Kumar P, Chacchi R, Murino A, Despott EJ, Lemmers A, Pioche M, Bourke M (2023) Duodenal neuroendocrine tumors: short-term outcomes of endoscopic submucosal dissection performed in the Western setting. Endosc Int Open 11(11):E1099–E1107 38026782 10.1055/a-2181-0320 PMC10681807

[32] Pavel M, Öberg K, Falconi M et al (2020) Gastroenteropancreatic neuroendocrine neoplasms: ESMO Clinical Practice Guidelines for diagnosis, treatment and follow-up. Ann Oncol 31:844–860 32272208 10.1016/j.annonc.2020.03.304

[33] Casali PG, Blay JY, Abecassis N et al (2022) Gastrointestinal stromal tumours: ESMO-EURACAN-GENTURIS Clinical Practice Guidelines for diagnosis, treatment and follow-up. Ann Oncol 33:20–33 34560242 10.1016/j.annonc.2021.09.005

[34] Serrano C, Martín-Broto J, Asencio-Pascual JM et al (2023) 2023 GEIS Guidelines for gastrointestinal stromal tumors. Ther Adv Med Oncol 15:17588359231192388 37655207 10.1177/17588359231192388 PMC10467260

[35] He G, Wang J, Chen B et al (2016) Feasibility of endoscopic submucosal dissection for upper gastrointestinal submucosal tumors treatment and value of endoscopic ultrasonography in pre-operation assess and post-operation follow-up: a prospective study of 224 cases in a single medical center. Surg Endosc 30(10):4206–4213. 10.1007/s00464-015-4729-1 26823060 10.1007/s00464-015-4729-1

